# Effectiveness comparisons of traditional Chinese medicine for psoriasis: A Bayesian network meta-analysis

**DOI:** 10.1097/MD.0000000000017055

**Published:** 2019-09-06

**Authors:** Longjun Wang, Yun Tao, Sheng Deng, Liyuan Chu, Linge Li

**Affiliations:** a Hebei University of Chinese Medicine; b Department of Dermatology, Huabei Petroleum General Hospital; c Department of Dermatology, Dongzhimen Hospital Affiliated to Beijing University of Traditional Chinese Medicine; d Shijiazhuang traditional Chinese Medicine Hospital, China.

**Keywords:** a network meta-analysis, protocol, psoriasis, Traditional Chinese medicine

## Abstract

**Background:**

Psoriasis is an immune-mediated polygenic hereditary skin disease quality of the patients‘ life because of the great trouble it causes to patients. Whereas, there is variability when we regard the selection of traditional Chinese medicine treatments in practice and most choices are made based on personal experience or preference of clinician. This study uses network meta-analysis to compare the effectiveness of different forms of TCM for psoriasis and assesses the evidence with the Grading of Recommendations Assessment, Development, and Evaluation (GRADE) approach.

**Methods:**

We will search for PubMed, Cochrane Library, AMED, EMbase, WorldSciNet; Nature, Science online and China Journal Full-text Database (CNKI), China Biomedical Literature CD-ROM Database (CBM), and related randomized controlled trials included in the China Resources Database. The time is limited from the construction of the library to April 2019. The quality of the included RCTs will be evaluated by the risk of bias (ROB) tool and the evidence will be evaluated by GRADE. STATA 13.0 and WinBUGS 1.4.3 through the GeMTC package will be used to perform a network meta-analysis to synthesize direct and indirect evidence.

**Results:**

The results of this network meta-analysis (NMA) will be submitted to a peer-reviewed journal for publication.

**Trial registration number:**

PROSPERO CRD42019137250.

## 1 Introduction

Psoriasis is a chronic inflammatory skin disease characterized by erythema, papules, and scaly lesions on the skin.^[1,2]^ The recurrence rate is high. The disease has a long course and repeated attacks. Both men, women, and children can have a genetic predisposition: if one of the parents has a history of psoriasis, the prevalence of the child is 16%. If both parents have a history of psoriasis, the child's chance of suffering from psoriasis increases. Up to 50%, for twins, if one person has psoriasis, the other party is fraternal twins, the other side is 23%, and if it is identical, the other party's prevalence increases to 63%.^[3]^ The disease is seasonal, with many winters and heavy summers, but some patients can be reversed, and the seasonality is not obvious after several years. At present, the incidence of psoriasis is yearly increasing, which seriously affects the quality of patients‘ lives because of the great trouble it causes to patients.^[4]^ According to reports, the incidence of psoriasis accounts for 0.1% to 3% of the world-wide population.^[5]^ A survey in the United States in recent years has shown that its incidence is 2.6%, which means an account of as many as 6 to 7 million.^[6,7]^ The cause and mechanism of psoriasis are still being explored. Yet, recently, it has been recognized that psoriasis is controlled by multiple genes and is also affected by factors such as heredity, infection, metabolism and immune dysfunction. Besides, seasonal changes, long-term wetlands, mental stimulation or trauma, surgery, etc. may induce or exacerbate the disease.^[8,9]^ Because of its stubbornness and high recurrence rate, curing psoriasis has long been a major mission in the world-wide medicine.

Psoriasis belongs to the category of “Baibi” in traditional Chinese medicine. Traditional Chinese medicine refers to this disease as “Baibi” and “pine bark lichen”.^[10–13]^ The Chinese medical literature also records the names of “skin–skin sputum”, “dry sputum”, “snake scorpion”, “white shell sore”, etc. Chinese medicine believes that the disease is caused by blood loss, dryness, and skin dystrophy.^[14,15]^ The spotted bleeding spots can be seen by scratching the scales, such as the name of the dagger that stabs the skin. Yin and Yang disorders of Zang and Fu organs are the basic pathogenesis of this disease.^[16,17]^ At present, Chinese medicine practitioners are constantly enriching and developing the etiology and pathogenesis of psoriasis. In the repeated and diverse clinical practice, modern medical doctors have a more detailed understanding of the etiology and pathogenesis of this disease, and to guide clinical treatment, and have achieved considerable therapeutic effects in the treatment of psoriasis. TCM has long history, significant clinical effects, and numerous relative clinical reports.^[18]^ However, the experimental design and quality of these studies are mixed with good and evil. To some degree, it affects the reliability of the research conclusions which makes the research results difficult to be recognized by the medical community. This study conducted a meta-analysis of published literature based on current research status and we expect to use a network meta-analysis to evaluate the efficacy and safety of TCM in the treatment of psoriasis in order to provide clues for clinical application and research.

## 2 Methods

This is a systematic review and ethical approval is not necessary.

### 2.1 Study registration

This systematic review protocol has been registered on PROSPERO as CRD42019137250. (http://www.crd.york.ac.uk/PROSPERO/display_record.php?ID=CRD42019137250)

### 2.2 Eligibility criteria

#### 2.2.1 Type of study

Randomized controlled trials of TCM (electroTCM, fire needle, plum blossom needle, TCM, embedding) or TCM combined with other effective interventions (drugs or other) as treatment methods, and the control group (effective methods other than TCM) must be exist. The language is limited to Chinese and English. Non-randomized controlled trials, quasi-randomized controlled trials, case series, case reports, crossover studies will be excluded.

#### 2.2.2 Participants

The patient must be at least 18 years of and less than or equal to 65 years of age. Gender is not restricted. The stage or severity of the disease is not limited. Psoriasis must be diagnosed according to at least one internationally or nationally authorized diagnostic criteria. The international standard refers to the diagnostic criteria for psoriasis in the “Cecil Textbook of Medicine”. Domestic standard refers to the diagnostic criteria for psoriasis in “Skins and Venereology”, “Clinical Dermatology” or “Integrated Chinese and Western Medicine Skin Dermatology”. The groups were well-balanced when they were enrolled.

#### 2.2.3 Types of interventions

##### 2.2.3.1 Experimental interventions

The drug composition, the dose-specific Chinese medicine preparation or the combined western medicine are used as experimental interventions. Both prescription and Chinese patent medicines will be included. Other traditional Chinese medicine treatments, such as intravenous medication, acupuncture, and massage will be limited.

##### 2.2.3.2 Control interventions

As for the control interventions, who accepted simple western medicine can be used as a control intervention or did not get any treatment as a blank control would be adopted. However, once they had accepted the therapy of TCM, the trials will be rejected.

#### 2.2.4 Outcomes

The primary outcome measurement is based on the psoriasis area and severity index (PASI) scoring criteria.

1. Healing: The rate of decline in PASI score after treatment was >90%.
2. Markedly effective: the rate of decline in PASI score after treatment is 60% to 89%.
3. Effective: The decline rate of PASI score is 20% to 59%.
4. Invalid: The decline rate of PASI score is <20%. PASI score decline rate = (pre-treatment PASI score − post-treatment PASI score) / pre-treatment PASI score × 100%. Total effective rate = (number of recovery cases + number of effective cases + number of effective cases) / total number of cases 100%.

The second outcome measure is based on TCM syndrome evaluation criteria.

(1) Healing: The clinical symptoms and signs of TCM disappear or almost disappear, and the syndrome score is reduced by ≥90%;
(2) Significant effect: The clinical symptoms and signs of TCM are obviously improved, and the syndrome score is reduced by ≥60%;
(3) Effective: Chinese medicine Clinical symptoms and signs have improved, syndrome scores decreased by <60%, but ≥30%;
(4) Invalid: The clinical symptoms and signs of TCM were not improved, even worse, and the syndrome score was reduced by < 30%. Integral variation formula (Nimodipine method: [(pre-treatment score - post-treatment score) ÷ pre-treatment score] × 100%.

#### 2.2.5 Data source

Database Search: PubMed, Cochrane Library, AMED, EMbase, WorldSciNet, Nature, Science online and China National Knowledge Infrastructure (CNKI), China Biomedical studies CD-ROM Database (CBM), China Resources Database. A studied review of clinical studies on TCM (or TCM) for the treatment of psoriasis published in domestic and foreign biomedical journals from the establishment of the library to April 2019. Based on the standards of the Cochrane Collaboration Workbook of the International Evidence-Based Medicine Center, a combination of manual searching and computer-based retrieval will be used to search relevant studies. The search terms include “Chinese medicine”, “traditional Chinese medicine”, “proprietary Chinese medicine”, “Chinese herbal medicine”, “psoriasis”, and “sub-sputum”. The search term in the Chinese database is the translation of the above word. The complete PubMed search strategy is summarized in Table 1.

**Table 1:**
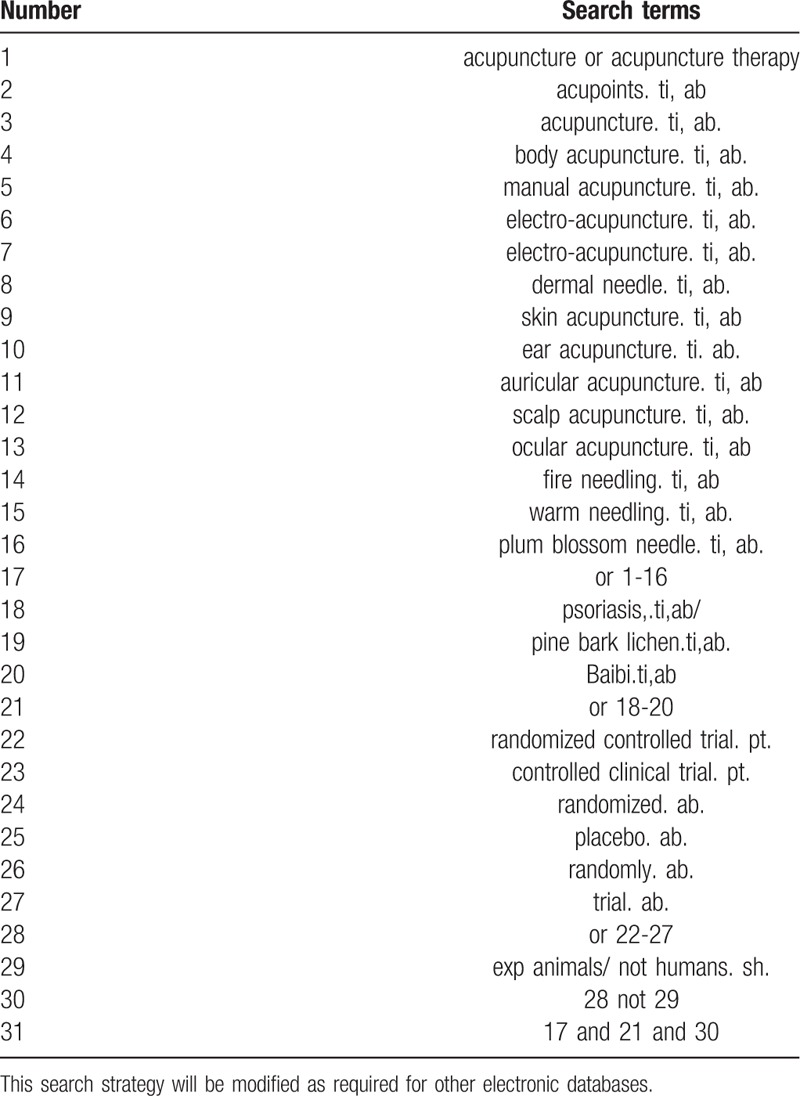
Search strategy used in PubMed database.

#### 2.2.6 Study selection

**Figure 1:**
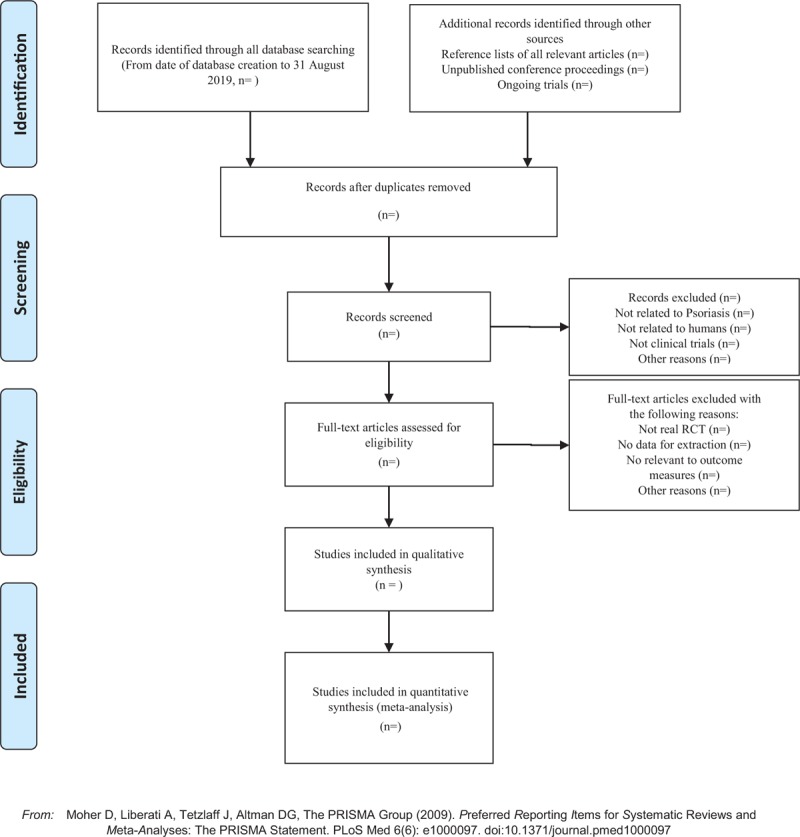
The PRISMA flow chart.

Applying the EndnoteX7 software to manage the included references. Two qualified evaluators independently screened the titles and abstracts of the selected studies, excluding duplicates and documents that did not significantly conform to the study. After a preliminary evaluation, the selected documents will be read one by one. Exclusions were based on inclusion criteria for uncontrolled studies, no randomization, inconsistent assessment criteria, and similar data. If there were different opinions, the third reviewer should be consulted. Studies information and data extraction were carried out on the final included studies, including the experimental methods of the study, the basic information of the included cases, the observation period, the intervention methods, observation indicators and test results of the treatment group and the control group. (Fig. 1)

#### 2.2.7 Risk of bias

The quality of the studies will be assessed by using the Cochrane Handbook 5.1.0 (Cochrane Handbook 5.1.0). The assessment will include random sequence generation, randomization correctness, allocation scheme hiding, blinding of patients and implementers, accuracy of data results, and other risk of bias. The risk of low bias is expressed as “low risk” and the risk of high bias is expressed as “high risk”. The information provided in the studies is inaccurate or does not provide sufficient information for the bias assessment to be expressed as “unclear risk”. The above content evaluation was independently evaluated by two researchers, and any differences will be resolved through discussions with the third reviewer.

#### 2.2.8 Statistical analysis

##### 2.2.8.1 Pairwise meta-analysis

The numerical variable will be expressed as the standardized mean difference (SMD) with a confidence interval (CI) of 95%. The heterogeneity of each pairwise comparison will be tested by chi-square test (test level α = 0.1). If there were no heterogeneity, a fixed effect model would be used. If there were significant heterogeneity between a group of studies, we would explore the reasons for the existence of heterogeneity from various aspects such as the characteristics of the subjects and the degree of variation of the interventions. Sensitivity analysis or meta-regression and subgroup analysis to explore possible sources of heterogeneity if it is necessary. We will use qualitative analysis of the funnel plot and graph symmetry to assess publication bias. Quantitative methods such as Begg testing and Egger testing will be used to help assess publication bias in the application.

##### 2.2.8.2 Network meta-analysis

STATA 13.0 and WinBUGS1.4.3 through the GeMTC package will be used to perform NMA to synthesize direct and indirect evidence. The NMA will be undertaken primarily in WinBUGS using the Markov chain Monte Carlo (MCMC) method.^[19]^ Convergence of the simu-lations will be evaluated with potential scale reduction factor (PSRF) and Gelman-Rubin-Brooks plots.^[20]^ The selection of the final model will depend on the deviance information criterion (DIC) value. Generally, a model with a smaller DIC value is better.^[21]^ Numerical variables will be presented as standardized mean differences (SMD) with 95% credible intervals (Cr I). The rank of treatments for each outcome will be conducted as surface under the cumulative ranking curve (SUCRA). The evidence relationship of included studies will be figured out by STATA. If there is a “closed loop,” the node splitting method will be used to evaluate the inconsistency of each loop.^[22,23]^

##### 2.2.8.3 Quality of evidence

The GRADE method will also be used to assess the quality of evidence for key outcomes. This assessment will be conducted through a Guideline Development Tool. (GRADEpro GDT, https://gradepro.org/)

## 3 Discussion

Psoriasis is an immunological skin disease characterized by T lymphocyte-mediated hyperproliferation of keratinocytes. Its etiology and pathogenesis have been extensively studied domestic and overseas, yet it is still unclear.^[24,25]^ Psoriasis has been reported all over the world. Due to the distinction of ethnicity, geographical location and environment, the prevalence of different populations exists great difference. The disease is prone to recurrence and has a long course of disease, and it is more likely to invade young adults. The disease has serious impacts on the health and mental state of patients and it is one of the refractory skin diseases.^[26]^ With the deep understanding of psoriasis and its complications, the trials and clinical reports of Chinese medicine treatment of psoriasis have been gradually increasing. TCM has the characteristics of small side effects, simple and easy to use, and has long been used to treat immune skin diseases. TCM therapy mainly achieves the therapeutic effect by stimulating the body's righteousness and regulating the balance of qi and blood.^[27]^ Although many studies have evaluated the effectiveness of TCM in the treatment of psoriasis, there is still a lack of evaluation and comparison of various treatments. To the best of our knowledge, NMA has not been used in recent years to compare the effectiveness of TCM in the treatment of psoriasis. The results of NMA can provide a possible ranking for TCM treatment of psoriasis. In addition, we will use the GRADE method to assess the quality of evidence for key outcomes. We hope that the results will provide clinicians with the best options for treating psoriasis and provide them with research directions. Although we will conduct a comprehensive search in this study, languages other than Chinese and English will be limited, which will lead to some bias. In addition, the literature on TCM treatment of psoriasis has a small sample size and low overall quality, which may affect the authenticity of this study. Therefore, the author hopes that in the future, there will be more rigorous and reasonable multi-center randomized controlled trials to explore the clinical efficacy of TCM treatment of psoriasis, so that assure the objectivity and rationality of conclusion.

## Author contributions

**Conceptualization:** Sheng Deng.

**Data curation:** Longjun Wang, Sheng Deng.

**Formal analysis:** Longjun Wang.

**Funding acquisition:** Liyuan Chu.

**Investigation:** Liyuan Chu.

**Resources:** Yun Tao.

**Software:** Yun Tao.

**Visualization:** Linge Li.

**Writing – original draft:** Linge Li.
